# Cholecystectomy for Biliary Colic Without Gallstones

**DOI:** 10.1155/1991/61926

**Published:** 1991

**Authors:** Christopher S. Worthley

**Affiliations:** Hepatobiliary and Pancreatic Surgical Unit Royal Adelaide Hospital Adelaide Australia


*HPB Surgery*, 1991, Vol. 4, pp 335–338
Reprints available directly from the publisher
Photocopying permitted by license only

© 1991 Harwood Academic Publishers GmbH
Printed in the United Kingdom

**HPB INTERNATIONAL**

**EDITORIAL & ABSTRACTING SERVICE**

JOHN TERBLANCHE, EDITOR
Department of Surgery,
Medical School · Observatory 7925
Cape Town · South Africa
Telephone: (021) 47-1250 Ext. 229
Telefax (021) 47-8955

### CHOLECYSTECTOMY FOR BILIARY COLIC WITHOUT GALLSTONES

**ABSTRACT**

*Gilliland, T.M. and Traverso, L.W. (1990) Cholecystectomy provides long-term symptom relief in patients with acalculous gallbladders. The American Journal of Surgery; 159: 489–492.*

**Elective cholecystectomy was performed in 60 patients with biliary colic and acalculous gallbladders during a 5-year period. Gallbladder wall disease was significantly more common ($p < 0.01$) in patients with acalculous gallbladders than in a similar symptomatic group with calculous gallbladders. Thirty-four of 43 patients (77%) available for long-term follow-up reported complete symptom relief after cholecystectomy. Preoperative ultrsonography, biliary scintigraphy, oral cholecystography, upper gastrointestinal series, and pathologic features of the gallbladder were equivocal in predicting long-term symptom relief. In patients with acalculous biliary colic, the best predictor of complete symptom relief is an adequate history of true biliary colic.**

### PAPER DISCUSSION

KEY WORDS: Cholecystectomy, gallstones, acalculous biliary colic

Historically, 30-75%[1,2] of patients with upper abdominal pain and an acalculous gallbladder respond to cholecystectomy. These differences in response are likely to be due to variations in selection criteria. It is these selection criteria which are debated by Gilliland and Traverso in the paper being reviewed.

The study attempted, retrospectively, to identify factors which predict longterm symptom relief in a group of 60 "acalculous" patients with persistent and intermittent right upper quadrant, or mid-epigastric pain, who underwent cholecystectomy. Symptom relief was obtained in 77% of the 43 patients followed up. The preoperative tests they described did not predict the patients likely to benefit. Nor did the presence of gallbladder pathology correlate with a successful outcome. The results were compared with those from another study[3] in which 650 patients with calculous disease were reviewed. Of particular interest was the observation that the preoperative detection of stones was not significantly more reliable in predicting cure than an adequate history of biliary colic.

This study suffers the deficiencies of all retrospective studies. However, it does throw down the gauntlet to proponents of new tests, asking that they show that such tests are superior to sound clinical judgement. Because of the likely multifactorial aetiology of acalculous gallbladder pain, it is difficult to develop a test which does not have a high false negative rate. These aetiological factors include gallbladder inflammation, cystic duct stenosis, microlithiasis, gallbladder ischaemia or dysmotility and sphincter of Oddi dysfunction. This disorder of biliary motility may be only one component of a more widespread "irritable bowel syndrome"[4,5].

Endoscopic retrograde choledochopancreatography (ERCP) is a test which was not found to be particularly useful in the study under review. In a larger series by Venu *et al.*[6], examining patients with normal gallbladder X-ray and ultrasound studies, ERCP was only beneficial in those with abnormal liver enzymes (25/32 benefited). When liver enzymes were normal, the yield was only 4/163 (2.5%).

Sphincter of Oddi manometry has principally been evaluated in the post-cholecystectomy pain patient. Hence, its significance in patients with acalculous biliary pain, normal liver enzymes and an intact gallbladder is uncertain. The detection of a potential sphincter abnormality may lead to sphincterotomy — a procedure usually more hazardous than cholecystectomy. So, to date, the principal role of manometry is in selected patients with continued symptoms after cholecystectomy.

Tests involving cholecystokinin (CCK) provocation have not been analysed by Gilliland and Traverso. These tests include pain reproduction[7] and gallbladder volume changes assessed by ultrasonography (US)[8], oral cholecystography (OCG)[8-11] or biliary scintigraphy[8,12,13]. Duodenal bile microscopy, looking for cholesterol crystals in the duodenum following CCK stimulation of the gallbladder, has also been assessed[9,14]. Sunderland and Carter[15] noted that where the use of one of these tests was supported, patients with negative tests had not undergone cholecystectomy. When the test under evaluation was not used to select patients for cholecystectomy, there did not seem to be a significant difference between test positive and negative patients[9-13,15]. Another criticism which can be levelled at earlier studies using CCK is that when given as a bolus, its effect is pharmacological, not reproducible, and influences intestinal as well as gallbladder motility. Hopman *et al.*[16] found that a constant infusion of CCK at 20ng/kg/hr produced a reliable and reproducible measure of gallbladder emptying in normal subjects. Biliary scintigraphy is likely to give a more reliable and simpler estimate of gallbladder volume than either US or OCG.

Thus, two recent reports of slow infusion CCK-stimulated cholescintigraphy in patients with acalculous biliary pain are of interest[17,18]. In the first study (Westlake *et al.*[17]), 69% of patients with chronic or recurrent acalculous biliary pain who underwent cholecystectomy improved. It was found that the gallbladder ejection fraction (GBEF) did not predict clinical outcome, the failure group having an even lower GBEF than the success group.

The second study (Yap *et al.*[18]) was designed differently in that patients with an "abnormal" GBEF were randomised to cholecystectomy or non-operative treatment. In the cholecystectomy group, 10/11 were cured. Conversely, all 10 in the non-operative group remained symptomatic. Thus, if the test was positive, the patient could be advised that their symptoms were likely to persist without cholecystectomy.

A negative test was observed in 82 (80%) patients. Sixty improved with time, usually after treatment of a non-biliary complaint. Eight were lost to follow-up while 14 underwent cholecystectomy for continued symptoms, eight (57%) being cured. The cure rate from cholecystectomy in this test negative group was not significantly different from that rate obtained when the test was positive (possibly reflecting a type II error). The principal advantage of a positive test seemed to be that treatment was expedited and the need for further investigation reduced.

These two studies[17,18] have conflicting conclusions which may reflect methodological differences and patient selection. In the first study[17], only those patients undergoing cholecystectomy were selected. In the second[18], it appears that patients were entered earlier in the diagnostic workup before a decision on cholecystectomy had been made.

It is therefore now important to see whether the test is also abnormal in patients with other conditions such as subacute small bowel obstruction, pancreatitis, severe duodenal ulceration and irritable bowel syndrome. Exclusion of such conditions in patients with a positive test may still be required before proceeding to cholecystectomy.

The advent of laparoscopic cholecystectomy forces us to re-evaluate the place of operative intervention in these patients[19]. The absence of detectable gallbladder stones and likely absence of dense adhesions make the patient with recurrent acalculous biliary pain ideally suited to this new technique. It is associated with lower morbidity, hospitalisation and time off work. In particular, the reduced local wound problems will improve the longterm success rate following cholecystectomy.

In summary, the need now exists for a prospective study of patients with typical acalculous biliary-type pain selected for laparoscopic cholecystectomy. They should be compared with a group of disease control patients who have upper abdominal pain due to other identifiable causes. Precise clinical details should be documented and biliary scintigraphy, as described by Yap *et al.*[18], should be performed in all patients with longterm follow-up being obtained. Although it appears that a prolonged GBEF may be as good a predictor of cure by cholecystectomy as is the detection of stones on ultrasound[3], the radionuclide test is relatively time-consuming, expensive and not readily available. It may be possible to reduce the need for such a test of gallbladder function by more closely examining the clinical features, as alluded to by Gilliland and Traverso. With selective use of biliary scintigraphy, it may be possible to expedite diagnosis and improve the eventual outcome following surgery.

### REFERENCES

1. Mackey, W.A. (1934) Cholecystitis without stones. *British Journal of Surgery*, **22**, 274–295
2. Gunn, A., Keddie, N.C. and Fox, H. (1973) Acalculous gallbladder disease. *British Journal of Surgery*, **60**, 213–215
3. Gilliland, T.M. and Traverso, L.W. (1990) Cholecystectomy: modern standards for comparison to alternative treatments for gallstones with emphasis on long-term symptom relief. *Surgery, Gynaecology and Obstetrics*, **170**, 39–44
4. Kellow, J.E., Miller, L.J., Phillips, S.F., Zinsmeister, A.R. and Charboneau, J.W. (1987) Altered sensitivity of the gallbladder to cholecystokinin octapeptide in irritable bowel syndrome. *American Journal of Physiology*, **253**, G650–655
5. Harvey, R.F. and Read, A.E. (1973) Effects of cholecystokinin on colonic motility and symptoms in patients with the irritable bowel syndrome. *Lancet*, **1**, 1–3
6. Venu, R.P., Geenan, J.E., Toouli, J., Stewart, E. and Hogan, W.J. (1983) Endoscopic retrograde cholangiopancreatography: diagnosis of cholelithiasis in patients with normal gallbladder X-ray and ultrasound studies. *Journal of the American Medical Association*, **249**, 758–761
7. Rhodes, M., Lennard, T.J.W., Farndon, J.R. and Taylor, R.M.R. (1988) Cholecystokinin (CCK) provocation test: long-term follow-up after cholecystectomy. *British Journal of Surgery*, **75**, 951–953
8. Davis, G.B., Berk, R.N., Scheible, F.W., Witztum, K.F., Gilmore, I.T., Strong, R.M. and Hofmann, A.F. (1982) Cholecystography, sonography and scintigraphy: detection of chronic acalculous cholecystitis. *Americal Journal of Roentgen*, **139**, 1117–1121
9. Burnstein, M.J., Vassal, K.P. and Strasberg, S.M. (1982) Results of combined biliary drainage and cholecystokinin in cholecystography in 81 patients with normal oral cholecystogram. *Annals of Surgery*, **196**, 627–732
10. Nora, P.F., Davis, R.P. and Fernandez, M.J. (1984) Chronic acalculous gallbladder disease: a clinical enigma. *World Journal of Surgery*, **8**, 106–112
11. Dunn, F.M., Christensen, E.C., Reynolds, J., Jones, V. and Fordtran, J.S. (1974) Cholecystokinin cholecystography. Controlled evaluation in the diagnosis and management of patients with possible acalculous gallbladder disease. *Journal of the American Medical Association*, **228**, 997–1003
12. Sunderland, G.T. and Carter, D.C. (1987) Cholecystokinin does not predict outcome in acalculous biliary pain. *British Journal of Surgery*, **74**, 1157 (abstract)
13. Pickleman, J., Peiss, R.L., Henkin, R., Salo, B. and Nagel, P. (1985) The role of sincalide cholescintigraphy in the evaluation of patients with acalculous gallbladder disease. *Archives of Surgery*, **120**, 693–697
14. Moskovitz, M., Min, T.C. and Gavaler, J.S. (1986) The microscopic examination of bile in patients with biliary pain and negative imaging tests. *American Journal of Gastroenterology*, **81**, 329–333
15. Sunderland, G.T. and Carter, D.C. (1988) Clinical application of the cholecystokinin provocation test. *British Journal of Surgery*, **75**, 444–449
16. Hopman, W.P.M., Jansen, J.B.M.J., Rosenbusch, G. and Lamers, C.B.H.W. (1986) Gallbladder contraction induced by cholecystokinin: bolus injection or infusion. *British Medical Journal*, **292**, 375–376
17. Westlake, P.J., Hershfield, N.B., Kelly, J.K., Kleiber, R., Lui, R., Sutherland, L.R. and Shaffer, E.A. (1990) Chronic right upper quadrant pain without gallstones: does HIDA scan predict outcome after cholecystectomy? *Americal Journal of Gastroenterology*, **85**, 986–990
18. Yap, L., Wycherley, A., Morphett, A. and Toouli, J. (in press) Acalculous biliary pain: cholecystectomy alleviates symptoms in patients with abnormal cholescintigraphy. *Gastroenterology*
19. Nottle, P.D., Wale, R.J. and Johnson, W.R. (1991) Percutaneous laparoscopic cholecystectomy: the first fifty. *Australian and New Zealand Journal of Surgery*, **61**, 254–260

Christopher S Worthley, FRACS
Hepatobiliary and Pancreatic Surgical Unit
Royal Adelaide Hospital
Adelaide, South Australia

